# Graph machine learning for integrated multi-omics analysis

**DOI:** 10.1038/s41416-024-02706-7

**Published:** 2024-05-10

**Authors:** Nektarios A. Valous, Ferdinand Popp, Inka Zörnig, Dirk Jäger, Pornpimol Charoentong

**Affiliations:** 1 Applied Tumor Immunity Clinical Cooperation Unit, National Center for Tumor Diseases (NCT), German Cancer Research Center (DKFZ), Im Neuenheimer Feld 460, 69120 Heidelberg, Germany; 2 Center for Quantitative Analysis of Molecular and Cellular Biosystems (Bioquant), Heidelberg University, Im Neuenheimer Feld 267, 69120 Heidelberg, Germany; 3 Division of Applied Bioinformatics, German Cancer Research Center (DKFZ), Im Neuenheimer Feld 280, 69120 Heidelberg, Germany; 4 Department of Medical Oncology, National Center for Tumor Diseases (NCT), Heidelberg University Hospital (UKHD), Im Neuenheimer Feld 460, 69120 Heidelberg, Germany

**Keywords:** Data integration, Computational science

## Abstract

Multi-omics experiments at bulk or single-cell resolution facilitate the discovery of hypothesis-generating biomarkers for predicting response to therapy, as well as aid in uncovering mechanistic insights into cellular and microenvironmental processes. Many methods for data integration have been developed for the identification of key elements that explain or predict disease risk or other biological outcomes. The heterogeneous graph representation of multi-omics data provides an advantage for discerning patterns suitable for predictive/exploratory analysis, thus permitting the modeling of complex relationships. Graph-based approaches—including graph neural networks—potentially offer a reliable methodological toolset that can provide a tangible alternative to scientists and clinicians that seek ideas and implementation strategies in the integrated analysis of their omics sets for biomedical research. Graph-based workflows continue to push the limits of the technological envelope, and this perspective provides a focused literature review of research articles in which graph machine learning is utilized for integrated multi-omics data analyses, with several examples that demonstrate the effectiveness of graph-based approaches.

## Background

Translational bioinformatics and data-driven biomedical research involving multi-omics profiling studies enable researchers to obtain comprehensive insights into key biological processes in health and disease. These studies are slowly becoming ubiquitous in biomedical research, and typically amalgamate genomics, epigenomics, transcriptomics, proteomics, metabolomics, metagenomics, and other modalities. Single-omic studies provide data and information pertinent to different functional and molecular layers. Single-omic approaches may lack the precision required to establish robust associations between molecular-level changes and phenotypic traits. Many diseases, including cancer, are the result of multistage processes and events that incorporate multiscale information from the genome to the proteome, consequently interactions and synergistic effects are much better explored through multi-omics analysis. Effectively, multi-omics experiments at bulk or single-cell resolution facilitate the discovery of hypothesis-generating biomarkers for predicting response to therapy, as well as aid in uncovering mechanistic insights into cellular and microenvironmental processes.

The primary motivation behind integrated data analysis is to identify key factors that explain or predict disease risk or other biological outcomes [1]. Integrated data derived from different sources are used for computational analysis through machine learning or biostatistics methods and eventually may lead to more solid results and output [2]. Many methods for data integration have been developed (concatenation-based, transformation-based, model-based, intermediate, hierarchical), all with strengths and weaknesses, and naturally, no single analysis approach will be optimal for all studies [1–3]. Furthermore, numerous integration strategies have been established involving graph-free workflows. For instance, these approaches—primarily based on the integration of genomics, epigenomics, transcriptomics, proteomics, and metabolomics data—have been utilized in, e.g., cancer research for the functional identification of driver genomic alterations, tumor classification, etc. [4]. In this paradigm, the multi-omics datasets are in a tabular format; rows represent samples and columns represent biological variables grouped by omics [3]. A simple concatenation of features across the omics (early integration) is likely to generate large matrices, outliers, and highly correlated variables [2]. A mixed integration strategy addresses the shortcomings of early integration by transforming independently each omics set into a simpler representation [3]. In intermediate integration, features are jointly integrated across the omics without prior omic-specific processing, with the advantage of processing features based on their redundancy or complementarity both within each omic and across the different omics [2]. Late integration is based on machine learning methods where a model is first trained for each omic to perform the prediction independently, and then the predictions achieved from each omic are combined via averaging or voting [2]. Machine learning models are commonly employed to analyze complex real-world data. In this context, unsupervised learning (e.g., dimensionality reduction) discovers patterns in multi-omics datasets without mapping input to output data [5, 6]. For example, variational autoencoders construct meaningful latent representations of integrated data, in an unsupervised way, by learning a compressed representation of the data and additionally by learning the underlying distribution parameters of the input data [7]. On the other hand, supervised learning, given input data and output labels, finds a function that maps the input to the label information (phenotypes of interest) [6]. Namely, Koh et al. developed a supervised learning method for integrating multi-omic profiles over genome-scale biological networks, and extracted network signatures predictive of pre-specified phenotypic groups [8]. Furthermore, mixed workflow approaches can be utilized for, e.g., modeling patient survival by processing multi-omics data via a combination of autoencoder and supervised machine learning algorithms [9].

In principle, all approaches aim to provide solutions for enhancing performance in a learning task, while mitigating, as much as possible, an array of challenges pertinent to data and methods. Methodologies commonly engage with incomplete, sparse, high-dimensional data, and obtain optimized representations and/or fuse information from multiple modalities. In this setting, some methods may tend to focus on a subset of modalities that are most helpful during model training while ignoring modalities that could be informative for model implementation, and because different modalities may lead to intricate relational dependencies, modality fusion may not fully leverage multimodal datasets [10]. In contrast, graph machine learning can model such datasets by connecting different modalities in optimally defined (but more realistically in context-defined) graphs, and by building learning systems for a wide range of tasks [10]. In this perspective, the authors are discussing the current trend of integrated multi-omics data analysis using graph machine learning approaches in the context of data-driven biomedical research.

## Graph modeling and machine learning

It is widely acknowledged that machine learning and especially deep learning systems have been very successful in analyzing complex biomedical datasets from a variety of domains and sources. Commonly, these datasets are defined in the Euclidean domain (modeled in an *n*-dimensional linear space, e.g., grid data) with existing deep learning methodologies developed to capture hidden patterns in such data, e.g., for large-scale image classification. Deep learning approaches using multi-omics datasets typically transform the high-dimensional features into high-level semantic embeddings, then learn a unified representation from the embeddings, and finally apply the learned representation for downstream tasks [11]. Hence, conventional deep learning approaches are rather limited in modeling the interrelationships/interactions among different omics, coupled with the incapacity to incorporate graph-based prior knowledge (e.g., protein-protein interaction networks) as input.

A different strategy for omics datasets would be to model them mathematically as graph-structured data, so that the relevant entities can be connected based on their intrinsic relationships, biological properties/significance, and empirical biomedical knowledge. All interactions within and across different omics sets form an interlinked graph (network) composed of vertices (nodes or entities) and edges (links or relationships). Effectively, omics information is no longer embodied as elements in data tables but rather as entities that are linked to one another by edges with properties/attributes that define the associations between the nodes. This heterogeneous graph representation of multi-omics (multiple types of nodes with diverse types of edges among them) provides an advantage for identifying patterns suitable for predictive or exploratory analysis, thus permitting the modeling of complex relationships and interactions.

Geometric deep learning encompasses emerging techniques that attempt to generalize structured deep neural models to graphs and manifolds [12]. Especially, graph machine learning methods have been developed to process data represented in the form of graphs, i.e., with an underlying structure that is a non-Euclidean space [13]. Graph neural networks (convolutional, attentional, message-passing) are performing inference over data embedded in a graph structure, consequently allowing for the learning process to consider the explicit relations of the data within and across different omics. Over the past few years, graph neural networks have become powerful and functional tools for machine learning tasks in the graph domain; this progress owes to advances in expressive power, model flexibility, and training algorithms [14]. On a practical note, there are several software libraries and tools that are regularly utilized for graph machine learning tasks; some of the more popular ones include: PyTorch Geometric (PyG) [15], Deep Graph Library (DGL) [16], Graph Nets [17], and Spektral [18]. Table 1 presents a broad categorization of graph machine learning techniques for multi-omics data. The table was adapted—from the general grouping of graph machine learning methods found in Xia et al. [19]—to reflect the multi-omics setting.

**Table 1:** Broad categorization of graph machine learning techniques for multi-omics data (adapted from Xia et al. [19]) with literature examples.

	References
Methods based on random walks	
They are useful for node classification and graph clustering. They simulate a process where a walker moves from one node to another in the graph by following edges randomly.	[43]
Methods based on matrix factorization	
They involve decomposing matrices associated with graphs into the product of two or more matrices, and are employed in miscellaneous graph-based learning tasks.	[44]
Methods based on deep learning	
They can learn representations and features from graph-structured data, e.g., graph autoencoders, graph convolutional networks, graph attention networks, and temporal graph networks.	[45–48]

Pertaining to the workings of a graph learning approach, a brief outline of the general framework of graph neural networks for node classification (supervised) is presented [20]. Let $G=(V,E)$ denote a graph where $V$ is the set of vertices or nodes and $E$ the set of the edges connecting the nodes [20]. Then, $A\in {{\mathbb{R}}}^{N\times N}$ represents the adjacency matrix where $N$ is the total number of nodes and $X\in {{\mathbb{R}}}^{N\times C}$ represents the node attribute matrix ($C$ is the number of features for each node) [20]. The objective is to learn effective node representations (denoted by $H\in {{\mathbb{R}}}^{N\times F}$ where $F$ is the dimension of node representations) by combining the graph structure information and the node attributes which are further used for node classification [20]. The essential idea of graph neural networks is to iteratively update the node representations by combining the representations of their neighbors and their own representations [20]. Starting from the initial node representation, ${H}^{0}=X$, in each layer there are two main functions: (1) AGGREGATE which aggregates information from the neighbors of each node, and (2) COMBINE which updates the node representations by combining the aggregated information from neighbors with the current node representations [20]. Therefore, the general framework of graph neural networks is defined by: Initialize: ${H}^{0}=X$; For $k={{{{\mathrm{1,2}}}}},\ldots ,K$; ${a}_{v}^{k}={{{{{{{\rm{AGGREGATE}}}}}}}}^{k}\left\{{H}_{u}^{k-1}:u\in N\left(v\right)\right\}$; ${H}_{v}^{k}={{{{{{{\rm{COMBINE}}}}}}}}^{k}\left\{{H}_{u}^{k-1},{a}_{v}^{k}\right\}$, with $N\left(v\right)$ being the set of neighbors for the $v$-th node [20]. The node representations ${H}^{K}$ in the last layer can be treated as the final node representations [20]. The computed node representations can be utilized for downstream tasks, e.g., node classification in which the label of node $v$ (denoted by ${\hat{y}}_{v}$) can be predicted through the Softmax function: ${\hat{y}}_{v}={{{{{{\rm{Softmax}}}}}}}\left(W{H}_{v}^{T}\right)$ where ${H}_{v}^{T}$ is the transpose of ${H}_{v}$ and $W\in {{\mathbb{R}}}^{\left|{\mathfrak{L}}\right|\times F}$ with $\left|{\mathfrak{L}}\right|$ being the number of labels in the output space [20]. Given a set of labeled nodes, the model can be trained by minimizing the loss function: $O=\left(1/{n}_{l}\right){\sum }_{i=1}^{{n}_{l}}{{{{{{\rm{loss}}}}}}}\left(\hat{{y}_{i}},{y}_{i}\right)$ where ${y}_{i}$ is the ground truth label of node $i$, ${n}_{l}$ is the number of labeled nodes, and ${{{{{{\rm{loss}}}}}}}\left(\cdot ,\cdot \right)$ is a loss function such as cross-entropy [20]. The whole model can be optimized by minimizing the objective function $O$ with backpropagation [20].

## Integrative analysis methodologies

**Fig. 1:**
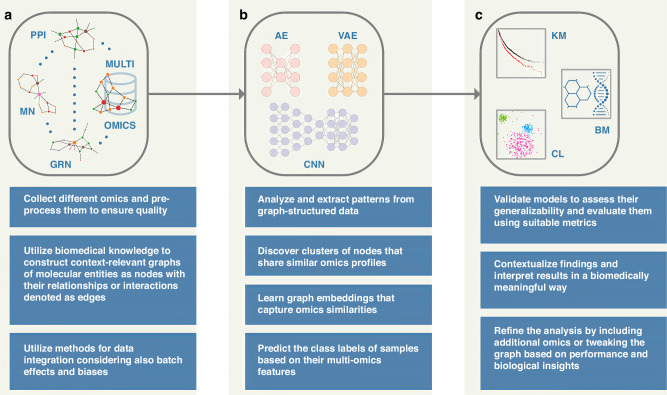
Conceptual workflow for integrated multi-omics analysis using graph machine learning approaches as a reliable methodological toolset in the context of precision medicine. Multiple modalities (**a**) such as genomics (somatic mutations, copy number variants, rare variants, genomic rearrangements, etc.), epigenomics (DNA methylation, chromatin accessibility, histone modifications, etc.), transcriptomics (mRNA expression, non-coding RNAs, etc.), proteomics (abundances and post-translational modifications), metabolomics (amino acids, organic acids, sugars, lipids, nucleotides, drugs, steroids, etc.), metagenomics (microbial enrichment, phylogeny, evolutionary profiles, etc.) and others are modeled as graph-structured data [2] along with prior knowledge such as, e.g., protein-protein interaction (PPI) networks, gene regulatory networks (GRN), and metabolic networks (MN). Graph machine learning methods [13] (**b**) are developed/applied for unsupervised, semi-supervised, and supervised learning [5, 6, 24] at the node, edge, or graph level for integrated analysis within and across different omics sets; these methods may include graph autoencoders (AE, upper left part), graph variational autoencoders (VAE, upper right part), and graph convolutional neural networks (CNN, lower part). The diagrams in (**b**) are generic architectural representations of the aforementioned neural networks. The overall objective is to translate the output into biomedical outcome (**c**): perform classification (e.g., tumor grade and subtype), form groups (patient clustering; CL), predict patient survival (KM), and identify potential biomarker (BM) candidates. The augmented information below each conceptual step of the workflow provides complementary details that correspond to general descriptions or actions that may fit different approaches. [Attribution: DNA/chemical formula vectors were adapted from vecteezy.com].

Multi-omics profiling technologies dive deeply into molecular landscapes and reveal multiple facets of complex research problems, e.g., shedding light on exciting novel aspects of cancer biology; these cutting-edge technologies produce large and intricate datasets, presenting researchers and clinicians with the considerable task of distilling complex information into clinical insights [21]. For instance, pan-cancer multi-omics analysis has revealed driver gene regulation via DNA methylation, offering insights into methylation-based stratification of cancer patients [22]. Accordingly, it is broadly acknowledged that there is a need for robust integrative analysis methodologies—for advancing precision medicine—that combine multiple data modalities effectively, hence taking into consideration the multilayered characteristics and interaction information of multi-omics datasets. Graph-based approaches—including graph neural networks—potentially offer a reliable methodological toolset that can provide a tangible alternative to scientists and clinicians that seek ideas and implementation strategies in the integrated analysis of their omics datasets for biomedical research. For example, graph convolutional networks can classify unlabeled nodes in a graph based on both their associated feature vectors as well as the network’s topology, making it possible to integrate graph-based data with feature vectors in a natural way [23]. Figure 1 shows a conceptual workflow for integrated multi-omics analysis using graph machine learning in the context of precision medicine, i.e., translating the output of these approaches into biomedical outcome.

A common approach when investigating graph-based methodologies is to model each omics dataset into a separate graph before analysis. Combining the separate graphs into a single homogeneous graph (through fusion) as the input of machine learning models allows for carrying out clustering, subtype discovery/classification, or survival prediction [3]. Furthermore, building a multilayered network with inter-layer connections (where each layer represents an omics set and interactions between omics sets are either inferred or retrieved from databases) allows for several methods to explore the network’s topology including shortest paths and random walks [3]. Graph embedding methods learn low-dimensional representations of nodes and their surroundings from each graph; the new graph-based features are then fed to machine learning models for prediction or classification [3]. This paradigm—graph representation learning—has emerged as a prominent machine-learning strategy for graphs, where the learned embeddings of graph elements are generated such that they capture the structure and semantics of the graph along with any downstream supervised task [24]. Graph representation learning includes methods for shallow graph embeddings that are utilized for node- and edge-property prediction, as well as graph neural networks that can generate representations for any graph element by capturing structure, attributes, and node metadata, thus utilized for node-, edge-, and graph-property prediction [24].

## Graph-based integrated multi-omics analysis

Graph-based workflows—including graph machine learning pipelines—continue to push the limits of the technological envelope, facilitating new investigations by combining patient information and biomedical knowledge. Several research articles showcase the potential of graph-based methodologies for integrated multi-omics analysis, hence offering scientists and clinicians inspiration and hints for tackling their elaborate research problems.

### Multi-omics integration with no network-based prior knowledge

Pai et al. presented a patient similarity graph-based approach for supervised patient classification using data (clinical; mRNA, miRNA, and protein expression; DNA methylation; and somatic copy number alteration) [25] from The Cancer Genome Atlas (TCGA) [26]. The authors demonstrated parity or superiority, comparing to other machine learning approaches (e.g., diagonal discriminant analysis, *k*-nearest neighbors, logistic regression, nearest centroid, partial least squares, random forests, and support vector machines) in predicting survival across four different tumor types, while visualizing the decision boundary in the context of patient similarity space thus making the results more interpretable [25]. As a further example, the authors compared their approach to another multi-omic patient classifier (e.g., DIABLO) showing that both tools provide complementary views of predictive multi-omic features that could be useful when applied in tandem [25]. A comparable approach showed that integrative modeling using genomics and electronic health record data has clinical utility [27]. Fang et al. showcased the effectiveness of a marginalized graph autoencoder for learning patient similarity feature representations followed by graph spectral clustering, in order to stratify non-small cell lung cancer patients into subgroups with distinct immunotherapy outcomes [27]. The authors explored differences in biological insight comparing their approach to the conventional log-rank test using clinico-genomic features, and indicated the potential of their method to inform insight on patient stratification as a complement to the traditional approach [27]. Wang et al. utilized labeled omics datasets (TCGA) and proposed a supervised multi-omics (mRNA and miRNA expression; and DNA methylation) integration approach based on deep multi-view learning (each omics data type as a particular view of the samples) [28]. The authors utilized graph convolutional networks for omics-specific learning, and a view-correlation discovery network to explore cross-omics correlations at the label space for effective multi-omics integration [28]. The authors compared the classification performance of their approach with other supervised multi-omics integration algorithms (e.g., *k*-nearest neighbors, support vector machines, lasso, random forests, gradient boosted trees, shallow and deep fully connected neural networks, adaptive group-regularized ridge regression, and two partial least squares discriminant analysis variants); their method outperformed the other methods in most classification tasks. Further ablation studies showed that their approach outperformed its variations in various classification tasks, and comparisons using their method on different omics sets showed that models trained with multi-omics data achieved better performance compared to single-omics models [28]. Overall, the authors successfully demonstrated their approach on tumor grade classification in low-grade glioma, kidney cancer type classification, and breast invasive carcinoma subtype classification, as well as biomarker identification related to breast cancer [28]. Likewise, Li et al. also developed a multi-omics integration model based on graph convolutional networks using copy number variation (exome-seq), transcriptomics (RNA-seq), proteomics (reverse-phase protein array), and clinical data from patients (TCGA) for cancer subtype analysis [29]. The authors applied a multi-modal autoencoder model to extract features and employed a similarity network fusion model to construct a patient similarity network; they compared their autoencoder with conventional methods such as principal component analysis, factor analysis, independent component analysis, and singular value decomposition [29]. Next, the authors utilized a graph convolutional network to integrate these two types of heterogeneous features and train the subtype classification model; they compared their graph convolutional network with methods such as decision trees, *k*-nearest neighbors, Gaussian naïve Bayes, random forests, support vector machines, a deep neural network with four layers, Grassmann clustering, and high-order path elucidated similarity [29]. Their method performed well for heterogeneous data integration, while addressing the issue of clinical interpretability [29]. Focusing on the interpretability aspect of graph convolutional neural networks explaining individualized predictions, Chereda et al. generated explanations in the form of relevant subgraphs for each data point, consequently providing interpretable molecular sub-networks that were individual for each patient [30].

### Multi-omics integration with network-based prior knowledge

In the context of integrating different multi-omics data and network-based prior knowledge [31], Kim et al. presented a graph-based semi-supervised framework for integrating multi-omics TCGA data (mRNA and miRNA expression; DNA methylation; and somatic copy number alteration) and genomic knowledge (pathway, gene ontology, motif, and chromosomal position gene sets), in an intermediate fashion, to predict outcomes according to survival, stage, and grade [32]. Data-driven graphs were generated from the multi-omics data and knowledge-driven graphs were generated from the genomic knowledge sets [32]. Their results suggested that the use of genomic knowledge improved the predictive power in explaining cancer phenotypes due to the synergies between genomic processes in the pathways involved in cancer [32]. The strengths of graph-based integration include its high computational efficiency (due to its sparseness properties) combined with an accuracy that is comparable to those of other methods such as kernel-based integration [32]. In a more recent approach [33], Ma and Zhang employed a multi-view factorization autoencoder to integrate multi-omics data (mRNA, miRNA, and protein expression; and DNA methylation) and protein-protein interaction (PPI) networks (STRING database [34]), learning feature and patient embeddings simultaneously. Their model can be used for unsupervised learning, but with available labeled data then supervised learning is possible by modifying the objective function [33]. The authors performed experiments on TCGA data for predicting the progression-free interval, and compared their model with other methods such as support vector machines, decision trees, naïve Bayes, random forests, AdaBoost, a variational autoencoder, and an adversarial autoencoder [33]. Inherently, the authors demonstrated that multi-omics data significantly outperformed single-omics, and additionally they showed that incorporating domain knowledge (e.g., biological interaction networks) in their model improves its generalizability and reduces the risk of overfitting [33]. In an analogous work, Schulte-Sasse et al. developed an interpretable graph deep learning approach to predict cancer genes from large datasets (pan-cancer data from the TCGA) by combining multi-omics data (mutations, copy number changes, DNA methylation, and mRNA expression) together with protein-protein interaction networks [23]. Identification of cancer genes plays a crucial role in the development of precision oncology and cancer therapeutics [23]. Furthermore, interpretability is valuable for assessing the molecular origin of a gene to be associated with cancer, detecting potential artifacts, and increasing trust in the modeling approach [23]. Their methodology used multi-dimensional multi-omics node features as well as topological features of the protein-protein interaction network in the learning process [23]. The authors compared their approach with methods grouped into different categories: omics only (methods that use only omics features for training, e.g., random forests), network only (methods that use only the PPI network, e.g., DeepWalk with support vector machines, graph convolutional network, and PageRank), network and omics (methods that use both data types, e.g., DeepWalk with random forest features, and HotNet2 diffusion), and cancer specific (methods specifically tailored to the prediction of cancer genes, e.g., MutSigCV, and 20/20+) [23]. The authors successfully recognized highly mutated cancer genes and genes harboring other kinds of alterations (aberrant DNA methylation, differential expression), consistently outperforming previous methods [23].

### Single-cell multi-omics integration

Single-cell multi-omics permits the quantification of multiple modalities for fully capturing the perplexity of complex molecular mechanisms and cellular heterogeneity [35]. Current methods for integrating single-cell multi-omics data typically consider the cells relationship between the reference and query datasets but ignore the relationship among cells within each dataset [36]. In addition, multiple datasets of the same or different omics often have unpaired cells (due to single-cell sequencing techniques being still cell destructive) [36]. In this setting, Cao and Gao developed a method for triple-omics integration, integrative regulatory inference, and multi-omics human cell atlas construction over millions of cells [37]. The authors utilized public datasets (transcriptome through single-cell RNA sequencing; chromatin accessibility through single-cell ATAC sequencing; and DNA methylation through single-nucleus methylome sequencing and single-cell combinatorial indexing for methylation analysis assay), and they systematically benchmarked their approach with multiple popular unpaired multi-omics integration methods, e.g., Online iNMF, LIGER, Harmony, bindSC, Seurat, UnionCom, Pamona, and MMD-MA [37]. By combining omics-specific autoencoders with graph-based coupling and adversarial alignment, the authors presented a modular framework (graph-linked unified embedding) for integrating unpaired heterogeneous single-cell multi-omics data and inferring regulatory interactions simultaneously; benchmarks showed that their approach was robust, scalable, and extendable [37]. Gao et al. presented a model, using public datasets, for integrating single-cell multi-omics data (transcriptome through single-cell RNA sequencing; chromatin accessibility through single-cell ATAC sequencing; and protein expression through CITE-seq–cellular indexing of transcriptomes and epitopes by sequencing) based on graph convolutional networks [36]. The authors compared their approach with four integration algorithms, e.g., Seurat, LIGER, GLUER, and Pamona [36]. Their results, by applying the method on six datasets, showed that data can be integrated from multiple single-cell sequencing technologies, species, or different omics, outperforming other methods [36]. Ma et al. developed a heterogeneous graph transformer model, using public datasets, for cell-type-specific biological network inference from single-cell multi-omics data (modalities: single-cell RNA sequencing, CITE-seq, and single-cell ATAC sequencing) [35]; their model was hypothesis-free and did not rely on the constraints of gene co-expressions. The authors compared their approach to other tools such as the graph-based method of Cao and Gao [37], Seurat, MOFA+, Harmony, and TotalVI [35]. For each benchmarking tool, grid-search tests were applied to a combination of parameters such as the number of dimensions for cell clustering and clustering resolution [35]. Their approach learnt relations among cells and genes within both local and global contexts, and performed better than existing tools in cell clustering and biological network construction [35].

## Challenges and opportunities

Graph-based multi-omics data integration may enable the formation of context-relevant networks that can capture the relations and interactions between different entities, e.g., genes, proteins, metabolites, etc., hence potentially offering a systems-level understanding of cellular and microenvironmental processes. Integration may aid in comprehending the function of genes and proteins more thoroughly (functional setting) and as a result deliver useful insights into biological processes. There might be possibilities in unraveling the mechanisms underlying diseases by studying the relationships between different biological components. In this context, subtyping diseases based on multi-omics profiles may offer perspectives into etiology and progression. Integration may allow for a more personalized approach to biomedicine by taking into account individual variations in omics profiles, as well as contributing to the identification of predictive biomarker candidates pertinent to the effect of a therapeutic intervention, thus aiding in the optimization of targeted therapies. On the other hand, integrating multi-omics data involves the coalescence of information from different molecular levels and this may pose challenges. In general, assembling omics data together into a more complete story is challenging mainly due to the diversity in dataset size, the patterns of missing data and noise across different data types, and the correspondences among measurements from different technologies [1]. More specifically, challenges may include: an imbalance at the class or feature level, missing values during data acquisition resulting in datasets with partial information, a larger number of features compared to a smaller number of patients, data with different distributions or types due to utilizing different technologies, and noise manifested as mislabeled samples.

Graph machine learning may offer a robust framework for integrating and analyzing multi-omics data by: (1) incorporating omics sets into a unified model, (2) scaling relatively well (up to a limit) in relation to growing data complexity, (3) providing more interpretable model predictions, (4) taking into account the complex interrelationships among the different molecular entities, (5) allowing for prior network-based knowledge integration, (6) leveraging the topology of the graph thus inferring potential associations that may not be apparent with conventional methods, (7) handling data heterogeneity related to scale and distribution quite well, (8) learning embeddings that describe the structure and relationships of the data, and (9) analyzing patterns of connectivity among multi-omics sets. The challenges in utilizing graph machine learning models on omics sets need to be acknowledged as well when developing such algorithms for analyzing biological data. A common challenge is the effort and domain expertise required for the construction of the graph which needs to be adapted to the problem at hand, but also relevant so that it reflects the inherent biological relationships. Data related problems such as increased heterogeneity (formats and scales), noise, and incomplete information can complexify the development of graph learning models. Often, the size of multi-omics datasets can be very large thus developing scalable models can be quite challenging as well. In this context, the large omics sets can make model training computationally expensive, ergo efficiency is paramount given that computational resources are finite. In this regard, Table 2 shows the general advantages and disadvantages of graph neural networks.

**Table 2:** General advantages and disadvantages of graph neural networks.

Advantages
State-of-the-art performance; versatility for a wide range of data types; can capture complex relationships and dependencies; well-suited when data context and connectivity are important; enhanced capability for transfer learning; scale efficiency for relatively large datasets; learning that factors in local and global information; can manage both graph structure and node/edge features; can combine information from proximal nodes/edges as well as the global context; extensions can handle heterogeneous graphs with different types of nodes and edges.
Disadvantages
Mainly can engage with static graphs; limited resilience to graph perturbations with incomplete or noisy data; computationally expensive on large graphs requiring significant resources for training; large amount of annotated data are required for training; model may lose information about specific nodes due to over-smoothing; can be challenging to generalize well on large graphs; challenging to find balance between incorporating prior information and learning from data; limited explainability unless extended to produce interpretable representations.

## The way forward

Integrated multi-omics analysis possesses considerable promise for resolving the inherent complexities of biological systems. New toolsets are supporting the research community [38] to, e.g., represent biomedical knowledge in a user-friendly manner by building task-specific knowledge graphs that facilitate the navigation and analysis of complex information [39]. Further research in the field of graph machine learning for integrated multi-omics analysis may assist in unraveling the intricate molecular interactions across diverse biological systems, which can pave the way for a more comprehensive understanding of disease and personalized therapeutic interventions. An increasingly popular and powerful self-supervised learning approach, for alleviating the reliance on labeled data, is contrastive learning [40]. This technique aims to learn salient features using raw input as the learning signal and usually leverages multiple positive and negative pairs of input samples in one batch, while substantial data augmentation is normally required for learning good and generalizable embedding features [40]. Extending contrastive learning to graph-structured data may improve performance for downstream analysis by, e.g., utilizing it for the pre-training of multi-omic graphical models. Furthermore, the spatial context in biological studies has profound biomedical/clinical relevance and implications. For instance, Hu et al. presented a graph convolutional network approach that integrated gene expression, spatial location, and histology to model the spatial dependency of gene expression for the identification of spatial domains and domain enriched spatially variable genes [41]. Going further, in order to develop a basic understanding of the molecular hierarchy from genome to phenome in individual cells, single-cell and spatial multi-omics methodologies (multimodal omics) are required [42]. In this paradigm, by leveraging the spatial information linking a cellular state to its respective micro- and macro-environments, through the use of graph neural networks, more fine-grained multimodal representations of cellular state should be obtainable [42].

## Conclusions

This perspective is focused on highlighting the significance of data-driven biomedical research, particularly within the context of integrating multiple omics. Integrated multi-omics analyses are crucial for exploring complex diseases, e.g., cancer, where multiple factors contribute to the disease’s development. Here, the use of machine learning is particularly emphasized for integrating and analyzing multi-omics datasets. Graph machine learning on heterogeneous omics sets has proven quite powerful as evidenced by previously published research in precision medicine, cancer biology, and other biomedical applications. Essentially, computational graph-based frameworks for bulk and single-cell integrated multi-omics analysis have indicated their capacity to clarify complex interrelationships and derive valuable insights from highly connected data. The different models presented have demonstrated that the enhanced capacity for analyzing omics interactions and the data integration with network-based prior knowledge are major advantages of graph-based approaches, with additional improvements in model generalizability. Certainly, there are issues on the data level as well as on the methodological level making integration a complicated endeavor when both are coupled for analyzing and in the end understanding complex biological systems. Nevertheless, factoring in the opportunities as well as the challenges of graph machine learning approaches on multi-omics data ensures for more sophisticated, adaptable, and refined models. These approaches will continue to provide demonstrable benefits to scientists and clinicians in terms of a more coherent and quantitative understanding of cell biology, but also more practically for improving the prediction of clinical outcome as well as assisting in the discovery of potential disease-related biomarker candidates.
